# High-density linkage map construction and QTL analyses for fiber quality, yield and morphological traits using CottonSNP63K array in upland cotton (*Gossypium hirsutum* L.)

**DOI:** 10.1186/s12864-019-6214-z

**Published:** 2019-11-21

**Authors:** Kuang Zhang, Vasu Kuraparthy, Hui Fang, Linglong Zhu, Shilpa Sood, Don C. Jones

**Affiliations:** 1 Crop & Soil Sciences Department, North Carolina State University, Raleigh, NC 27695 USA; 2 Cotton Incorporated, 6399 Weston Parkway, Cary, NC 27513 USA; 3 4 Cityplace drive, The Climate Corporation (Bayer U.S. Crop Science), St. Louis, MO 63141 USA

**Keywords:** Upland cotton, Single nucleotide polymorphism (SNP), Array, Breeding, Mapping, Recombinant inbred lines (RILs), Linkage map, Quantitative trait locus (QTL), QTL clusters, Fiber quality and yield

## Abstract

**Background:**

Improving fiber quality and yield are the primary research objectives in cotton breeding for enhancing the economic viability and sustainability of Upland cotton production. Identifying the quantitative trait loci (QTL) for fiber quality and yield traits using the high-density SNP-based genetic maps allows for bridging genomics with cotton breeding through marker assisted and genomic selection. In this study, a recombinant inbred line (RIL) population, derived from cross between two parental accessions, which represent broad allele diversity in Upland cotton, was used to construct high-density SNP-based linkage maps and to map the QTLs controlling important cotton traits.

**Results:**

Molecular genetic mapping using RIL population produced a genetic map of 3129 SNPs, mapped at a density of 1.41 cM. Genetic maps of the individual chromosomes showed good collinearity with the sequence based physical map. A total of 106 QTLs were identified which included 59 QTLs for six fiber quality traits, 38 QTLs for four yield traits and 9 QTLs for two morphological traits. Sub-genome wide, 57 QTLs were mapped in A sub-genome and 49 were mapped in D sub-genome. More than 75% of the QTLs with favorable alleles were contributed by the parental accession NC05AZ06. Forty-six mapped QTLs each explained more than 10% of the phenotypic variation. Further, we identified 21 QTL clusters where 12 QTL clusters were mapped in the A sub-genome and 9 were mapped in the D sub-genome. Candidate gene analyses of the 11 stable QTL harboring genomic regions identified 19 putative genes which had functional role in cotton fiber development.

**Conclusion:**

We constructed a high-density genetic map of SNPs in Upland cotton. Collinearity between genetic and physical maps indicated no major structural changes in the genetic mapping populations. Most traits showed high broad-sense heritability. One hundred and six QTLs were identified for the fiber quality, yield and morphological traits. Majority of the QTLs with favorable alleles were contributed by improved parental accession. More than 70% of the mapped QTLs shared the similar map position with previously reported QTLs which suggest the genetic relatedness of Upland cotton germplasm. Identification of QTL clusters could explain the correlation among some fiber quality traits in cotton. Stable and major QTLs and QTL clusters of traits identified in the current study could be the targets for map-based cloning and marker assisted selection (MAS) in cotton breeding. The genomic region on D12 containing the major stable QTLs for micronaire, fiber strength and lint percentage could be potential targets for MAS and gene cloning of fiber quality traits in cotton.

## Background

The cotton genus *Gossypium* spp. consists of at least 51 species, with 45 diploid (2n = 2x = 26) and six allotetraploid (2n = 4x = 52, AD) [1, 2] species. Of these only four are cultivated species: *G. hirsutum* L. (2n = 4x, AADD), *G. barbadense* L. (2n = 4x, AADD), *G. arboreum* L. (2n = 2x, AA) and *G. herbaceum* L. (2n = 2x, AA). *G. hirsutum* L., also called Upland cotton, contributes to more than 90% of the global cotton production and acreage and *G. barbadense* L., known as Pima cotton, accounts for 8% of the cotton production in the world [3].

As the largest natural fiber source, cotton is one of the most important economic crops worldwide. In 2018/19 season, cotton was primarily grown in around 30 countries, with more than 116 million bales of fiber produced [4]. In the United States, which is the third largest cotton fiber producing country as well as the largest cotton fiber exporting country in the world, 18.59 million bales of cotton fiber was produced with 15 million bales exported in 2018/19 season [4]. The production, distribution and processing of cotton in the United States provide about $27 billion direct business revenue while supporting more than 200 thousand jobs [5]. However, the world cotton fiber market is recently under a lot of pressure because of the development of synthetic fibers [6]. In addition, the US cotton has to compete with handpicked cotton from Asia. Currently, the US cotton could compete in the international markets because of its higher fiber quality. Therefore, improving the fiber quality has been an important objective of cotton breeders in the US. Farm productivity and economic viability of cotton production directly related to the lint yields [5]. As such, continued improvements in the fiber quality and yield are critical for the US cotton production.

Plant height, a typical quantitatively inherited trait [7–9], can indirectly influence the yield of cotton fiber because optimal plant height can contribute to machine harvesting and help achieve higher harvesting index [7]. Fuzziness seed trait, an important seed trait related to the cotton yield and fiber quality [10], was usually considered as a binomial trait (fuzzy seed or fuzzless seed) while some reports indicated this trait was polygenically controlled [10–13].

In general, fiber quality and yield traits in cotton are known to inherit polygenically and influenced by environment [14–16]. Further, fiber quality traits often have negative association with some yield traits [17]. Although, traditional breeding methods played an important role in the development of cotton cultivars [18, 19], further improvements in the trait values especially for the quantitative traits using these breeding approaches have been limited [20, 21]. With the advancement of molecular marker technology, maker-assisted selection (MAS) has been increasingly applied in the cotton breeding programs [22]. Restriction fragment length polymorphism (RFLP) markers were the first type of the markers used in the cotton improvement [23] and the first linkage maps in cotton were constructed using RFLP markers in 1994 [24]. From then on, various types of the molecular markers were used in the cotton genetics and breeding [25–32]. High-density genetic maps with broadly adaptable markers are required for improving the efficiency in detection and MAS-based transfer of quantitative trait loci (QTLs) [33–39]. The abundance, extensive polymorphism and compatibility to high-throughput genotyping platforms have made the single nucleotide polymorphism (SNP) markers the most popular markers used in plant translational genomics [40–42]. With the development of next-generation sequencing (NGS) technologies, several methods to discover large numbers of SNP-based markers are now developed for cotton [36–40]. This enabled the development of high-density linkage maps in cotton [36–40]. In the present study, we used 63K SNP array [40] for genotyping a recombinant inbred line (RIL) population, derived from landrace by elite germplasm line cross, to construct a high-density linkage map and to map the QTLs for cotton fiber quality, yield and morphological traits in Upland cotton.

## Results

### Analyses of the phenotypic traits

A summary of the statistical analyses for the phenotypic performance of the twelve traits is presented in Table 1. Among the six fiber quality traits measured, micronaire (MIC), upper half mean length (UHM), uniformity index (UI) and fiber strength (STR) of the parental accession NC05AZ06 were significantly (*P* < 0.05) higher (13.0–16.9%, 34.1–36.6%, 4.4–7.6%, 7.4–8.1%, respectively) than those of the parental accession NC11–2091 while the short fiber content (SFC) of NC11–2091 was significantly (*P* < 0.05) greater (26.3–55.3%) than that of NC05AZ06. No significant difference was found between the two parents for the fiber elongation (ELO). All the four yield traits, boll weight (BW), lint percentage (LP), seed index (SI) and lint index (LI) were significantly (*P* < 0.01) higher (209.4–222.8%, 137.2–160.0%, 12.5–24.6%, 311.8–317.9%, respectively) in NC05AZ06 than in NC11–2091. For morphological traits, the plant height (PH) of NC05AZ06 was significantly (*P* < 0.01) lower (− 32.5%) than NC11–2091. The seed fuzziness grade (FG) of NC05AZ06 was 100% (fuzz-rich) and the FG of NC11–2091 was 0 (fuzz-free). The broad-sense heritability of the traits calculated by the ratio of total genetic variance to total phenotypic variance for all the traits is listed in Table 2. Most traits, except for PH, had high broad-sense heritability across 2 years with values ranging from 82 to 96%. The broad-sense heritability of PH was only 56%. Since we only had 1 year’s data for PH, we can just state that the trait performance of PH might be sensitive to the environment.

**Table 1:** Phenotypic trait performance of the RIL population and their parents evaluated in the field at Central Crops Research Station, Clayton, NC in years 2016 and 2017

Type of phenotype	Phenotypic Trait^b^	Year	Parents			RILs			
			NC05AZ06 (P1)	NC11–2091 (P2)	P1-P2	Min	Max	Mean	SD
Fiber Quality	MIC (μg/inch)	2016	4.90	4.19	0.71^b^	3.69	7.02	4.81	0.55
		2017	4.78	4.23	0.55^c^	3.65	6.20	4.79	0.46
	UHM (Inches)	2016	1.12	0.82	0.3^c^	0.73	1.11	0.92	0.08
		2017	1.14	0.85	0.3^c^	0.74	1.13	0.93	0.1
	UI (%)	2016	82.25	76.43	5.82^b^	73.93	83.95	79.36	1.6
		2017	83.20	79.73	3.48^c^	76.95	84.40	81.62	1.5
	STR (g/tex)	2016	27.64	25.56	2.08^b^	22.31	32.57	26.82	2.13
		2017	27.45	25.55	1.9^b^	20.90	30.55	25.49	2.23
	ELO (%)	2016	6.92	6.74	0.19	3.85	12.54	7.26	1.28
		2017	8.00	8.60	−0.6^b^	4.60	12.50	8.81	1.41
	SFC (%)	2016	8.32	12.92	−4.61^b^	7.16	17.90	10.63	1.67
		2017	8.35	10.55	−2.2^c^	6.85	17.10	9.05	1.61
Yield component-related	BW(g)	2016	4.92	1.59	3.33^c^	1.46	4.23	2.77	0.6
		2017	6.23	1.93	4.3^c^	2.03	5.61	3.46	0.75
	LP(%)	2016	40.8	17.2	23.6^c^	16.39	39.22	28.63	4.98
		2017	40.3	15.5	24.8^c^	19.00	38.50	28.21	4.44
	SI (g)	2016	10.19	8.18	2.01^c^	7.13	11.70	9.13	0.82
		2017	11.15	9.91	1.24^c^	8.14	12.90	10.54	0.93
	LI (g)	2016	7.00	1.70	5.3^c^	1.86	5.62	3.69	1.06
		2017	7.48	1.79	5.69^c^	2.31	6.58	4.18	1.18
Morphological	PH (cm)	2017	44.3	65.6	−21.3^c^	32.15	66.25	48.04	4.95
	FG (%)	2016	100	0	100^c^	0	100	66.7	38.2
		2017	100	0	100^c^	0	100	43.8	37.9

^a^ *MIC* micronaire, *UHM* upper half mean length, *UI* uniformity index, *STR* fiber strength, *ELO* fiber elongation, *SFC* short fiber content, *BW* boll weight, *LP* lint percentage, *SI* seed index, *LI* lint index, *PH* plant height, *FG* fuzziness grade of seed

^b^ 0.05 significance level; ^c^ 0.01 significance level

**Table 2:** The broad-sense heritability of fiber quality, yield component related and morphological traits in the RIL population evaluated in the field at Central Crops Research Station, Clayton, NC across 2 years (2016 and 2017)

	MIC^a^	UHM	UI	STR	ELO	SFC	BW	LP	SI	LI	PH^b^	FG
V_g_	0.230	0.0077	2.507	4.593	1.675	3.004	0.430	21.032	0.797	1.197	24.517	15.60
V_p_	0.258	0.0084	3.003	5.255	1.811	3.534	0.522	21.958	0.931	1.257	43.422	17.26
H^2^	89%	92%	83%	87%	92%	85%	82%	96%	86%	95%	56%	90%

The broad-sense heritability (H^2^) = genetic variance (V_g_)/phenotypic variance (V_p_)

^a^ *MIC* micronaire, *UHM* upper half mean length, *UI* uniformity index, *STR* fiber strength, *ELO* fiber elongation, *SFC* short fiber content, *BW* boll weight, *LP* lint percentage, *SI* seed index, *LI* lint index, *PH* plant height, *FG* fuzziness grade of seed

^b^ PH with only year 2017 data used

The results of correlation analyses for the twelve traits was described in Table 3. Among the fiber quality traits, UHM was significantly (*P* < 0.01) positively correlated with UI, BW, LP, LI, FG, and significantly (*P* < 0.01) negatively correlated with MIC, ELO and SFC. The STR was significantly positively correlated with BW (*P* < 0.05), SI (*P* < 0.01) and PH (*P* < 0.05), and was significantly negatively correlated with ELO (*P* < 0.05) and LP (*P* < 0.01). The SFC was significantly (*P* < 0.01) positively correlated to MIC, ELO and it was significantly (*P* < 0.01) negatively correlated to UI. The ELO was significantly (*P* < 0.01) positively correlated with MIC and significantly negatively related to UI (*P* < 0.01) and BW (*P* < 0.05) (Table 3). Almost all the four yield traits BW, LP, SI, and LI showed a highly positive correlation with each other, except for LP and SI, which the correlation was not significant (Table 3). The morphological trait PH had a negative correlation with yield traits BW, LP and LI, and a positive correlation with SI and STR, respectively. Another morphological trait fuzziness grade was highly positively correlated with all the four yield traits (Table 3).

**Table 3:** Correlation analysis between the phenotypic traits in the RIL population evaluated in the field at Central Crops Research Station, Clayton, NC across 2 years (2016 and 2017)

Trait	MIC^a^	UHM	UI	STR	ELO	SFC	BW	LP	SI	LI	PH^b^
UHM	−0.36^d^										
UI	−0.28^d^	0.82^d^									
STR	0.18	−0.12	−0.08								
ELO	0.3^d^	−0.62^d^	−0.51^d^	− 0.2^c^							
SFC	0.24^d^	−0.79^d^	− 0.93^d^	0.1	0.46^d^						
BW	0.12	0.29^d^	0.18	0.21^c^	−0.23^c^	− 0.13					
LP	0.28^d^	0.25^d^	0.11	−0.27^d^	0.09	−0.09	0.46^d^				
SI	−0.06	0.12	0.19^c^	0.32^d^	−0.17	−0.15	0.37^d^	−0.04			
LI	0.21^c^	0.3^d^	0.18	−0.1	0	− 0.14	0.61^d^	0.89^d^	0.4^d^		
PH	−0.06	−0.13	− 0.12	0.21^c^	− 0.11	0.11	− 0.25^c^	−0.36^d^	0.21^c^	−0.22^c^	
FG	−0.14	0.27^d^	0.2^c^	−0.09	−0.11	− 0.18	0.46^d^	0.31^d^	0.32^d^	0.42^d^	−0.2

^a^ *MIC* micronaire, *UHM* upper half mean length, *UI* uniformity index, *STR* fiber strength, *ELO* fiber elongation, *SFC* short fiber content, *BW* boll weight, *LP* lint percentage, *SI* seed index, *LI* lint index, *PH* plant height, *FG* fuzziness grade of seed

^b^ PH used only year 2017 data

^c^ 0.05 significance level; ^d^ 0.01 significance level

### Construction of linkage maps

**Fig. 1:**
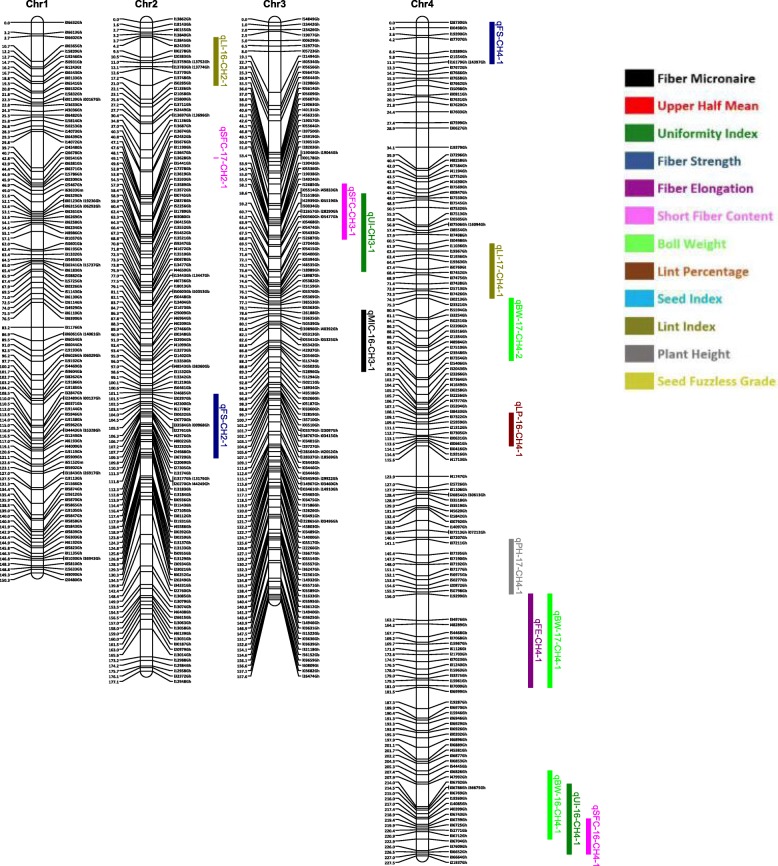
Linkage map for chromosomes Chr1(D9), Chr2(A13), Chr3(A3), Chr4(A11) along with the detected QTLs

**Fig. 2:**
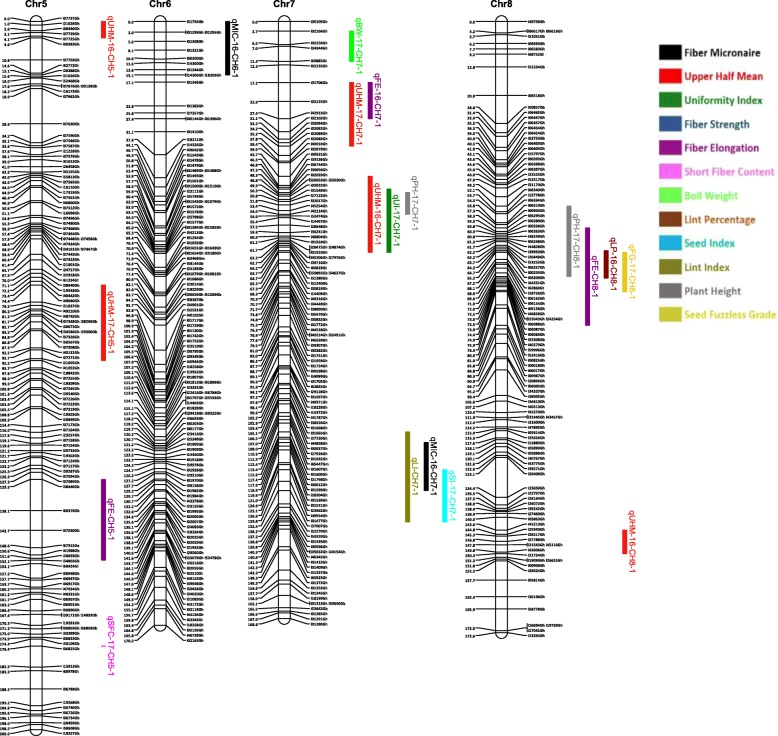
Linkage map for chromosomes Chr5(D11), Chr6(D7), Chr7(A7), Chr8(A9) along with the detected QTLs

**Fig. 3:**
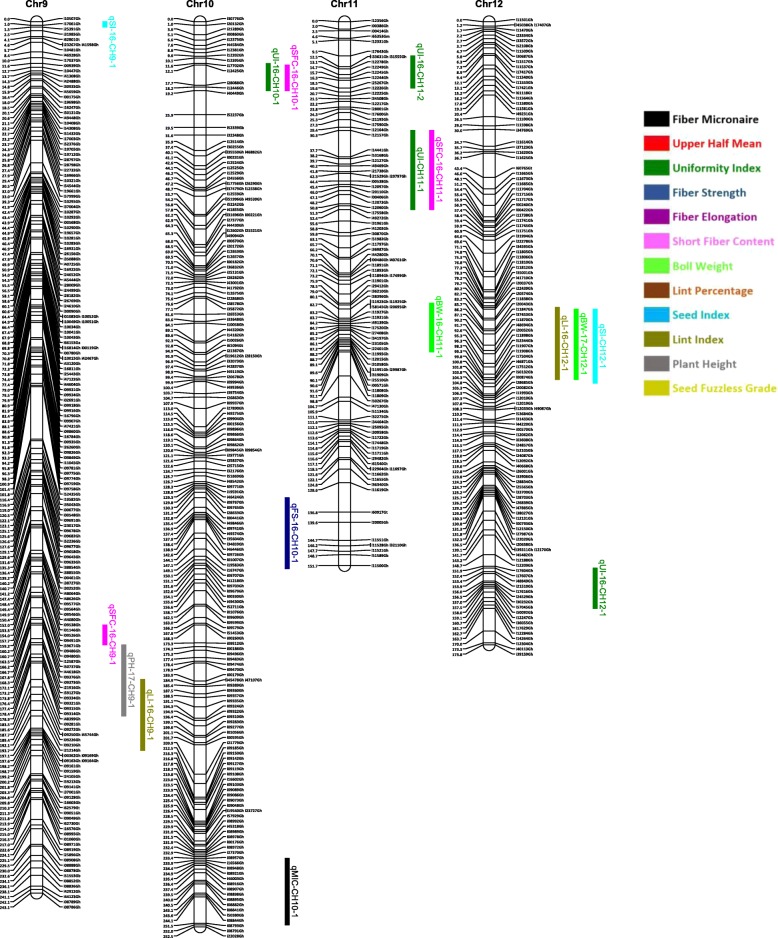
Linkage map for chromosomes Chr9(D5), Chr10(A5), Chr11(A10), Chr12(D10) along with the detected QTLs

**Fig. 4:**
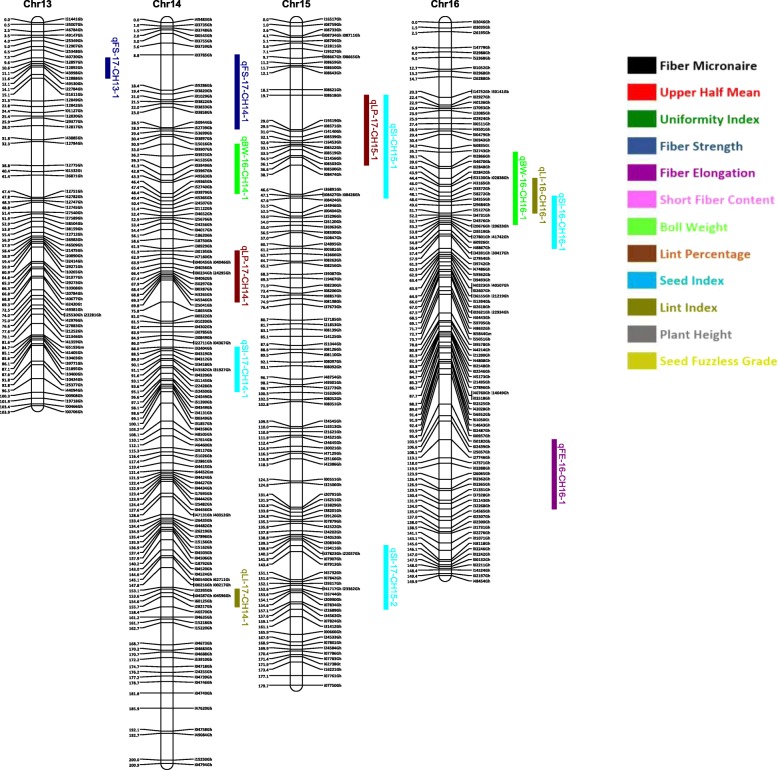
Linkage map for chromosomes Chr13(A4), Chr14(A8), Chr15(A12), Chr16(A1) along with the detected QTLs

**Fig. 5:**
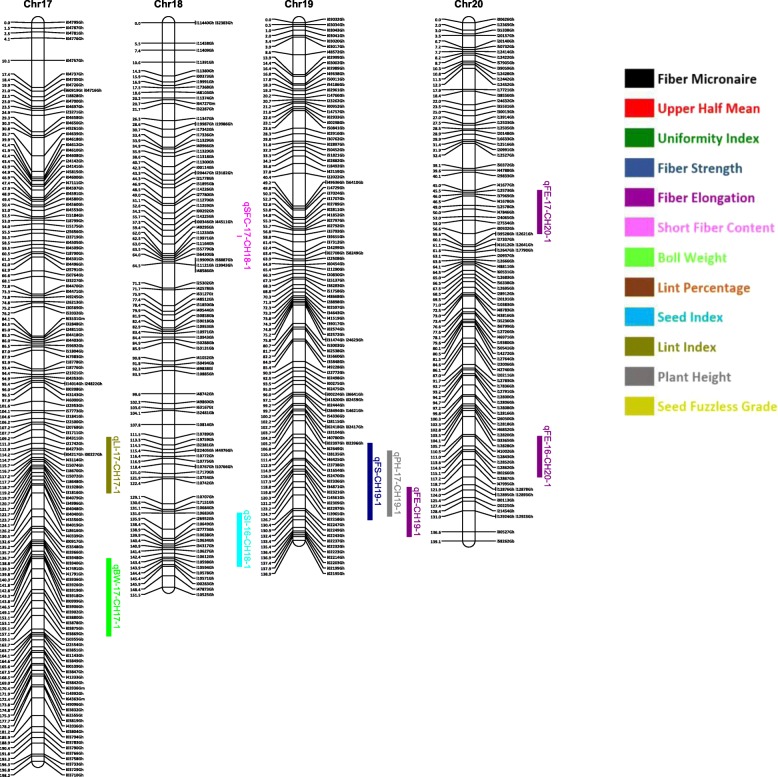
Linkage map for chromosomes Chr17(D8), Chr18(A6), Chr19(D1), Chr20(D4) along with the detected QTLs

**Fig. 6:**
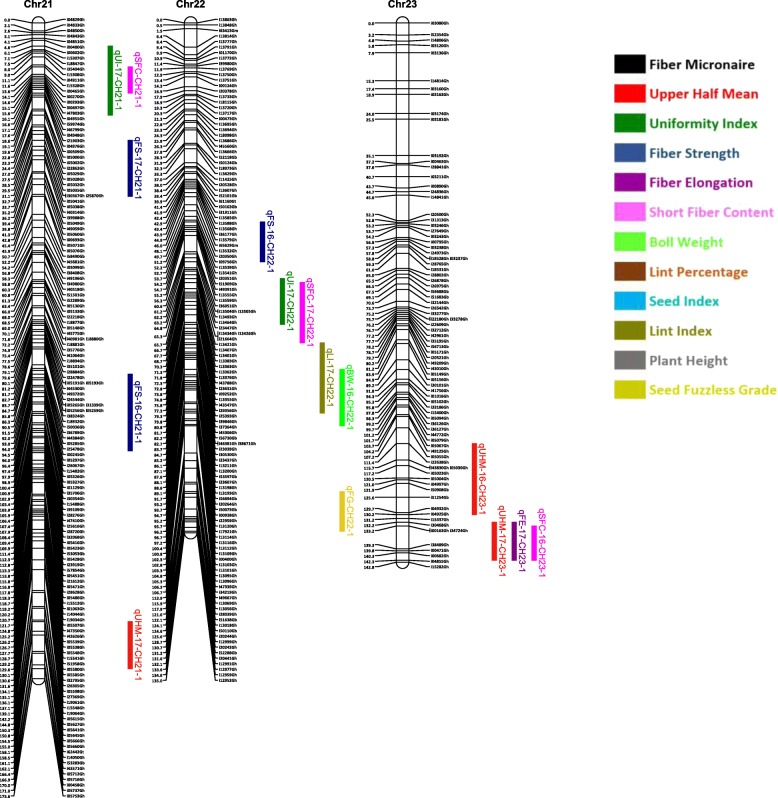
Linkage map for chromosomes Chr21(D2), Chr22(D13), Chr23(A2) along with the detected QTLs

**Fig. 7:**
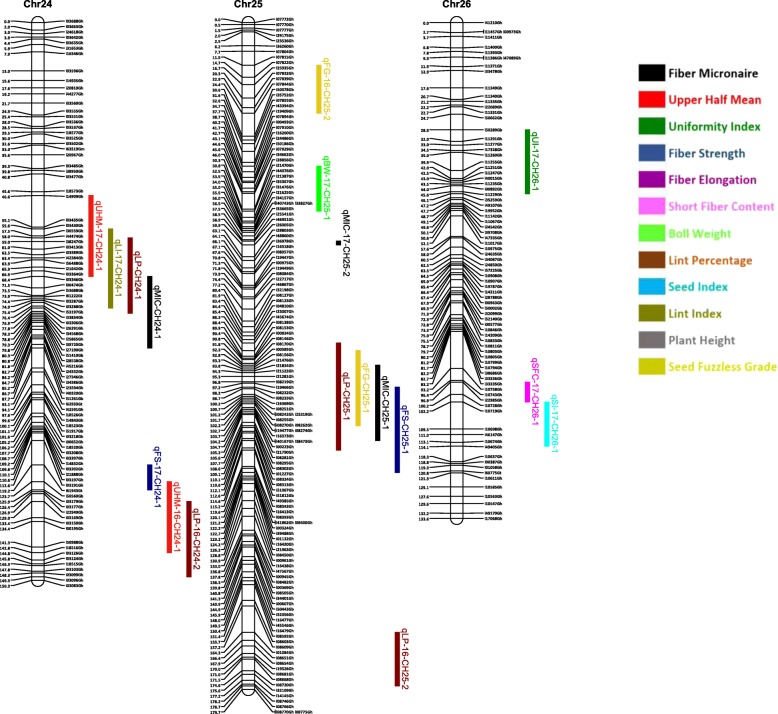
Linkage map for chromosomes Chr24(D3), Chr25(D12), Chr26(D6) along with the detected QTLs

Out of 63,058 SNPs used in the genotyping, 11,255 (17.8%) SNPs were polymorphic between the two parents. A total of 3129 SNPs were selected for linkage map construction after removing the poor quality or duplicate SNPs. All the 3129 markers were mapped on 26 linkage groups (26 chromosomes) (Figs. 1, 2, 3, 4, 5, 6 and 7, and Additional file 2: Table S2). This resulted in the genetic map length of 4422.44 cM with an average distance of 1.41 cM between markers (Table 4). Of these 3129 SNPs, 1534 SNPs were mapped to the A sub-genome while 1595 SNPs were mapped to the D sub-genome. The mapped SNPs of the A sub-genome generated a genetic map of 2236.35 cM with an average marker density of 1.46 cM while 1595 SNPs of the D sub-genome gave a genetic map of 2186.09 cM with an average marker density of 1.37 cM (Table 4). Genetic lengths of 26 linkage groups ranged from 103.9 cM to 252.5 cM. Number of markers mapped per chromosome range from 69 to 180 and average marker density ranging from 1.09 cM to 1.72 cM in each group (Table 4). Five gaps (adjacent marker distance > 10 cM) with the interval distances of 11.02 cM, 11.30 cM, 14.59 cM, 10.01 cM and 10.01 cM were identified on 5 different linkage groups Chr.03 (A3), Chr.08 (A09), Chr.09 (D5), Chr.26 (D6) and Chr.05 (D11), respectively (Table 4).

**Table 4:** Details of the linkage maps constructed using the Cotton 63 K SNP array and the RIL population of cross between NC05AZ06 and NC11–2091

Linkage group	Corresponding Chr.	No. of markers mapped	Genetic length (cM)	Avg. marker density	Gaps^a^ (> 10 cM)	No. of QTLs mapped	Distorted Markers^b^	No. of SDR^c^
Chr16	A1	110	149.9	1.36	0	4	3	1
Chr23	A2	83	142.82	1.72	0	4	2	0
Chr03	A3	139	157.56	1.13	1	3	13	1
Chr13	A4	69	103.9	1.51	0	1	0	0
Chr10	A5	180	252.5	1.4	0	4	5	0
Chr18	A6	100	151.52	1.52	0	2	1	0
Chr07	A7	115	168.56	1.47	0	9	6	0
Chr14	A8	130	200.94	1.55	0	5	3	0
Chr08	A9	109	172.61	1.58	1	5	6	1
Chr11	A10	104	151.72	1.46	0	4	12	1
Chr04	A11	149	227.53	1.53	0	10	4	0
Chr15	A12	112	179.68	1.6	0	3	13	2
Chr02	A13	134	177.11	1.32	0	3	34	3
A-Sub-genome		1534	2236.35	1.46	2	57	102	9
Chr19	D1	111	138.85	1.25	0	3	0	0
Chr21	D2	147	173.6	1.18	0	5	0	0
Chr24	D3	93	150.29	1.62	0	7	4	0
Chr20	D4	99	139.11	1.41	0	2	9	1
Chr09	D5	179	243.08	1.36	1	4	19	2
Chr26	D6	82	133.64	1.63	1	3	5	1
Chr06	D7	127	170.51	1.34	0	1	7	1
Chr17	D8	138	198.21	1.44	0	2	9	1
Chr01	D9	113	150.31	1.33	0	0	0	0
Chr12	D10	123	173.79	1.41	0	4	12	1
Chr05	D11	123	199.99	1.63	1	4	1	0
Chr25	D12	136	179.68	1.32	0	8	3	0
Chr22	D13	124	135.03	1.09	0	6	4	1
D-Sub-genome		1595	2186.09	1.37	3	49	73	8
Total		3129	4422.44	1.41	5	106	175	17

^a^Gap: Distance between two adjacent markers > 10 cM

^b^Distorted Markers: Markers showing segregation distortion (chi-square test *P* < 0.05)

^c^SDR segregation distortion region

Of the 3129 mapped SNPs, 175 (5.6%) SNP markers showed segregation distortion which spanned on 22 chromosomes, with the most distorted markers (34) and highest distortion rate (25.37%) on Chr.02 (A13) (Table 4). Seventeen segregation distortions region (SDR) were identified on 13 chromosomes, with 9 of the SDRs in A sub-genome and 8 SDRs in the D sub-genome (Table 4). Hence, the sub-genomes did not show any bias for the SDRs.

**Fig. 8:**
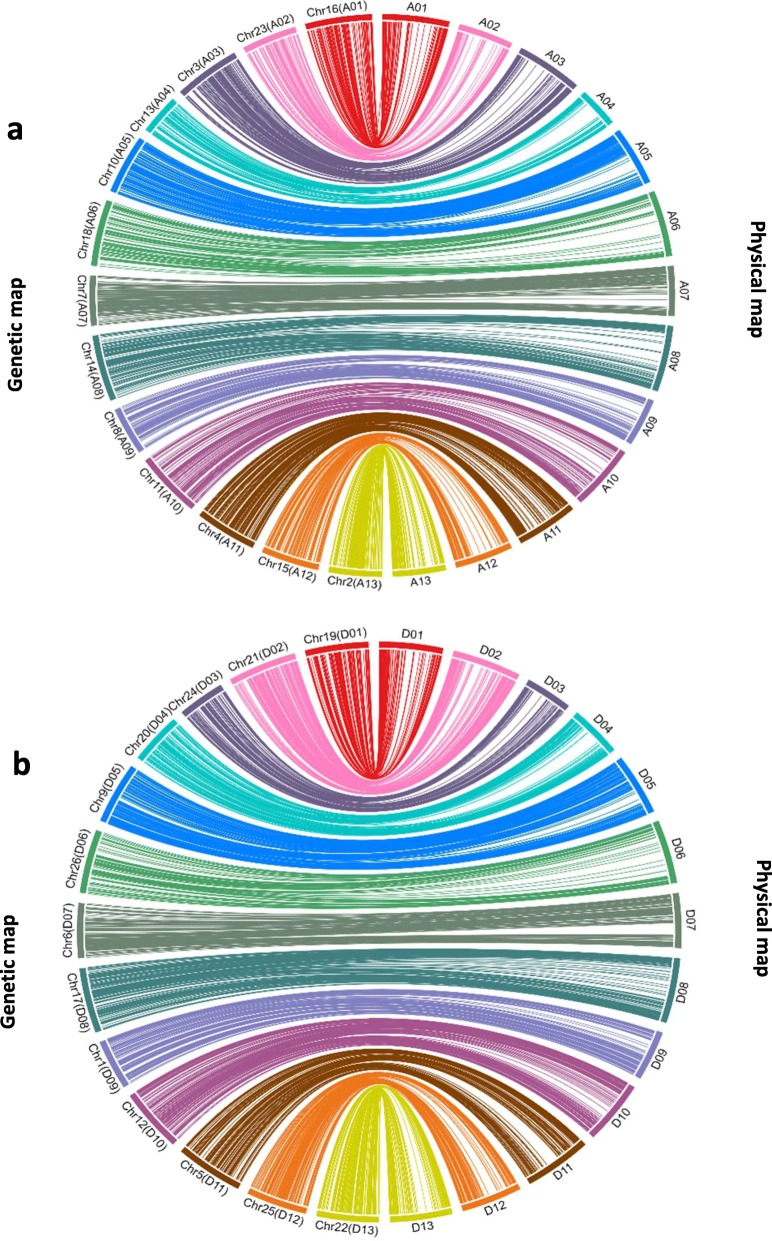
Collinearity between genetic map and physical maps of A sub-genome and D sub-genome of Upland cotton developed using recombinant inbred line mapping populations genotyped with 63 K SNP array. **a** Collinearity for chromosomes in A sub-genome. **b** Collinearity for chromosomes in D sub-genome

Comparison of the genetically mapped SNPs with the sequence based physical map of the TM-1 (*G. hirsutum*) reference genome sequence [43] for syntenic relationships showed that the strong collinearity between the genetic map and physical map (Fig. 8). The SNP based genetic map of 4422.44 cM corresponded to 1911.76 Mb of the sequence based physical map which represented 98.8% of the total length of the sequence based physical map (Additional file 2: Table S2 and Additional file 4: Table S4). All linkage groups showed good collinearity with the physical map. Coverage of the individual chromosomes ranged from 96.4 to 99.5% of the sequence based physical map. Figure 8 shows the circos plots that describe strong collinearity between the genetic map and physical map. Finally, collinearity between genetic and physical maps suggest that the genetic mapping population used in the current study did not contain any chromosomal rearrangements.

### QTL analysis for cotton fiber quality, yield and morphological traits

QTL analysis using composite interval mapping (CIM) identified a total of 106 QTLs, with 59 of QTLs for fiber quality traits, 38 for yield traits and 9 for morphological traits (Additional file 1: Table S1). Overall the phenotypic variation explained by the QTLs ranged from 3.6–48.0% (Additional file 1: Table S1). Among the 106 QTLs, 22 were stable QTLs identified in both years, 40 QTLs were identified only in 2016 and 44 QTLs were identified only in 2017. By determining that the SFC with lower value was favorable and other traits (BW, SI, LI, LP, STR, MIC, UHM and UI) with higher value were favorable, the favorable alleles of 80 QTLs were derived from NC05AZ06 (P1) with positive additive effects whereas 26 QTLs with negative additive effects were contributed by NC11–2091 (P2). Of the 106 QTLs, 57 QTLs were mapped in the A sub-genome and 49 QTLs were in the D sub-genome (Table 4). Among the 57 A sub-genome QTLs, 43 QTLs with favorable alleles were from NC05AZ06 and 14 were from NC11–2091. In the D sub-genome, 37 QTLs with favorable alleles were contributed by NC05AZ06 and the 12 were contributed by NC11–2091. Overall, of the 106 mapped QTLs, 46 QTLs were major QTLs with PVE > 10%. These included 29 QTLs for fiber quality traits (Table 5) (18 in the A sub-genome and 11 in the D sub-genome), 12 QTLs for yield traits (Table 6) (5 QTLs in the A sub-genome and 7 in the D sub-genome) and 5 QTLs for morphological traits (one in A sub-genome and 4 in D sub-genome (Table 7).

**Table 5:** Major^b^ QTLs for the fiber quality traits identified in the RIL population phenotyped at the Central Crops Research Station, Clayton, NC in years 2016 and 2017

Phenotypic Trait^c^	QTL	Year	Range	Peak	LOD	PVE^d^	AE^e^
MIC	qMIC-CH10-A5–1^a^	16	233.43–252.01	244.08	11.4	16.2	−0.25
		17	233.43–252.01	243.58	9.1	16.2	−0.207
	qMIC-CH24-D3–1^a^	16	71.47–87.7	81.76	16.3	25.8	0.335
		17	68.4–87.7	78.81	12.5	23	0.256
	qMIC-CH25-D12–1^a^	16	102.67–109.11	104.67	3.3	4.1	0.128
		17	92.32–112.58	102.67	6.1	10	0.167
	qMIC-16-CH3-A3–1	16	78.61–95.33	88.82	11.7	17.2	0.344
	qMIC-16-CH6-D7–1	16	0–15.09	3	7	19.3	0.274
UHM	qUHM-16-CH5-D11–1	16	0–4.58	0	6.5	11.2	−0.028
	qUHM-16-CH7-A7–1	16	43.66–65.19	55.42	6.9	12.1	0.029
	qUHM-17-CH7-A7–1	17	17.16–35.23	22.56	6.1	10.1	0.027
	qUHM-17-CH23-A2–1	17	132.16–142.33	142.33	6.6	10.2	0.028
	qUHM-16-CH24-D3–1	16	123.73–142.8	134.44	6.7	11.9	0.028
UI	qUI-CH3-A3–1^a^	16	46.82–68.03	58.14	10.4	21	−0.921
		17	46.82–68.03	55.53	3.5	6	−0.386
	qUI-CH11-A10–1^a^	16	30.34–52.37	42.28	8.4	16.1	0.9
		17	46–50.81	46	2.8	4.9	0.362
	qUI-16-CH4-A11–1	16	207.88–227.03	220.88	7.4	13	0.72
	qUI-16-CH10-A5–1	16	11.59–19.17	17.67	7.2	12.7	0.732
	qUI-17-CH21-D2–1	17	6.05–24.36	17.12	6.1	10	0.488
	qUI-17-CH26-D6–1	17	28.88–46.13	39.93	7	13.1	−0.596
STR	qSTR-CH2-A13–1^a^	16	107.73–118.93	112.84	4.9	7.3	0.639
		17	101.54–118.93	111.34	7.2	11.9	0.767
	qSTR-CH19-D1–1^a^	16	121.23–124.72	124.72	3.6	5.2	−0.519
		17	112.85–133.39	121.23	7.3	11.8	−0.726
	qSTR-CH25-D12–1^a^	16	98.22–115.15	108.63	9.4	15.6	−0.936
		17	101.68–121.15	110.59	7.3	11.5	−0.701
	qSTR-17-CH14-A8–1	17	8.8–28.94	21.46	7.5	11.9	0.736
ELO	qELO-CH4-A11–1^a^	16	156.02–172.58	167.72	4.7	7.1	−0.385
		17	156.02–181.46	172.58	8.7	13.5	−0.562
	qELO-CH8-A9–1^a^	16	58.29–74.89	68.12	7.7	12.3	0.507
		17	65.15–85.78	71.03	5.3	7.5	0.437
	qELO-CH19-D1–1^a^	16	124.72–137.87	136.4	7.7	12.4	0.499
		17	126.74–137.87	136.4	5.6	8.1	0.428
	qELO-17-CH23-A2–1	17	132.16–142.33	142.33	7	11.2	−0.503
SFC	qSFC-CH3-A3–1^a^	16	44.13–59.15	58.64	3.9	7.9	0.664
		17	46.82	46.82	2.5	18.4	0.807
	qSFC-17-CH2-A13–1	17	36.99	36.99	3.6	20.6	−0.837
	qSFC-16-CH4-A11–1	16	217.44–227.03	226.53	7.4	12.4	−0.811
	qSFC-17-CH5-D11–1	17	176.37	176.37	3.1	20.1	−0.828
	qSFC-17-CH18-A6–1	17	57.29	57.29	3.2	20.4	−0.827

^a^QTLs identified in both years. ^b^Major QTLs: The QTLs with at least 1 year’s PVE > 10%. ^c^MIC, micronaire; UHM, upper half mean; UI, uniformity index; STR, fiber strength; ELO, fiber. ^d^PVE, phenotypic variation explained. ^e^AE, additive effect

**Table 6:** Major^b^ QTLs of the yield traits identified in the RIL population phenotyped at the Central Crops Research Station, Clayton, NC in years 2016 and 2017

Trait^c^	QTL	Year	Range	Peak	LOD	PVE^d^	AE^e^
BW	qBW-16-CH4-A11–1	2016	204.27–222.9	214.51	9.3	14	0.217
	qBW-17-CH4-A11–1	2017	156.02–181.46	171.61	8.9	12.1	0.251
	qBW-16-CH22-D13–1	2016	91.66–106.73	91.66	7.8	12.4	0.212
LP	qLP-CH24-D3–1^a^	2016	58–78.32	66.92	11.2	16.6	1.885
		2017	58–76.37	65.94	7.3	8.8	1.208
	qLP-CH25-D12–1^a^	2016	86.34–105.64	97.24	4.5	5.9	1.077
		2017	97.24–115.15	107.65	12.5	17.7	1.666
	qLP-17-CH14-A8–1	2017	61.88–75.77	68.84	11.1	15.2	1.561
SI	qSI-CH12-D10–1^a^	2016	80.19–98.29	91.65	14.8	27.1	0.712
		2017	80.19–100.77	90.17	14.8	30.4	0.669
	qSI-CH15-A12–1^a^	2016	19.71–38.68	33.54	10.7	17.5	−0.432
		2017	19.71–47.61	38.68	6.7	10.6	−0.332
LI	qLI-16-CH2-A13–1	2016	4.21–17.19	11.03	7	10.8	0.297
	qLI-16-CH12-D10–1	2016	79.71–99.78	90.17	8.9	13.5	0.324
	qLI-17-CH22-D13–1	2017	84.67–103.29	94.72	13.7	21.1	0.437
	qLI-17-CH24-D3–1	2017	55.56–76.86	66.92	9.2	12.6	0.337

^a^QTLs identified in both years

^b^Major QTLs: The QTLs with at least one year’s PVE > 10%

^c^BS, boll weight; LP, lint percentage; SI, seed index; LI, lint index

^d^PVE, phenotypic variation explained

^e^AE, additive effect

**Table 7:** Major QTLs^b^ of the morphological traits identified in the RIL population phenotyped at the Central Crops Research Station, Clayton, NC in years 2016 and 2017

Trait^c^	QTL	Year	Range	Peak	LOD	PVE^d^	AE^e^
PH	qPH-17-CH8-A9–1	17	52.05–72.01	56.8	5.5	10.3	−2.557
	qPH-17-CH9-D5–1	17	173.76–193.74	185.58	7.9	15.8	−2.625
	qPH-17-CH19-D1–1	17	114.85–132.4	124.72	5.8	10.4	−2.204
FG	qFG-CH22-D13–1^a^	16	124.58–134.55	134.55	23.5	39.2	24.361
		17	124.09–134.55	134.55	28.4	48	29.28
	qFG-CH25-D12–1^a^	16	88.36–108.63	98.71	14.8	19	−15.112
		17	98.71–104.67	101.19	4	3.6	−7.972

^a^QTLs identified in both years

^b^Major QTLs: The QTLs with at least one year’s PVE > 10%

^c^PH, plant height; FG, fuzziness grade of seed

^c^PVE, phenotypic variation explained

^d^AE, additive effect

### QTL for fiber quality traits

A total of 59 QTLs, including 15 stable QTLs, 23 QTLs in 2016 and 21 QTLs in 2017, were identified for six fiber quality traits with the PVE ranging from 4.1 to 25.8% (Table 5, Additional file 1: Table S1). Parental accession NC05AZ06 contributed favorable alleles for 43 QTLs while NC11–2091 donated 16 QTLs. Sub-genome wide, of the 59 fiber quality QTLs, 31 QTLs were mapped in the A sub-genome (24 QTLs with favorable alleles from NC05AZ06 and 7 from NC11–2091) and 28 QTLs were mapped on the D sub-genome (19 QTLs with favorable alleles from NC05AZ06 and 9 from NC11–2091).

#### Micronaire (MIC)

For fiber micronaire, seven QTLs explaining 4.1 to 25.8% of the phenotypic variance (PV) were identified, among which 5 are major QTLs (Table 5 and Additional file 1: Table S1). Three major stable QTLs, qMIC-CH10-A5–1, qMIC-CH24-D3–1, and qMIC-CH25-D12–1 explained 16.2–16.2%, 23–25.8%, 4.1–10.0% of phenotypic variance, respectively. Two major QTLs qMIC-16-CH3-A3–1 and qMIC-16-CH6-D7–1 with the PVE 17.2 and 19.3%, respectively, were detected in the 2016 dataset. The qMIC-CH10-A5–1 was the only QTL with favorable alleles derived from parental accession NC11–2091.

#### Upper half mean length (UHM)

UHM is a measure of fiber length. Ten QTLs explaining 5.5 to 12.1% of PV were identified (Table 5 and Additional file 1: Table S1). Five major QTLs, including 3 QTLs (qUHM-16-CH5-D11–1, qUHM-16-CH7-A7–1, qUHM-16-CH24-D3–1) in 2016 and 2 QTLs (qUHM-17-CH7-A7–1, qUHM-17-CH23-A2–1) in 2017, with the PVE ranging from 10.1 to 12.1% were detected. Majority of the QTLs with favorable alleles were derived from the parent NC05AZ06. The qUHM-16-CH5-D11–1 was the only QTL with favorable alleles derived from NC11–2091.

#### Uniformity index (UI)

Ten QTLs explaining 4.9 to 21% of PV were detected and mapped for UI in the genetic maps (Table 5 and Additional file 1: Table S1). Seven QTL favorable alleles were conferred by parental accession NC05AZ06. Of these, six were major QTLs. These included 2 stable QTLs, qUI-CH3-A3–1 and qUI-CH11-A10–1 with 6.0–21.0%, 4.9–16.1%, respectively, of PVE and 4 single-year QTLs (qUI-16-CH4-A11–1, qUI-16-CH10-A5–1, qUI-17-CH21-D2–1, qUI-17-CH26-D6–1) explaining 10.0–13.1% of PV.

#### Fiber strength (STR)

For fiber strength, 11 QTLs explaining 4.1 to 15.6% of PV, with 7 QTLs having favorable alleles conferred by NC05AZ06 were detected (Table 5 and Additional file 1: Table S1). Of these, four were major QTLs, including three stable QTLs (qSTR-CH2-A13–1, qSTR-CH19-D1–1, qSTR-CH25-D12–1) with PVE of 7.3–11.9%, 5.2–11.8%, 11.5–15.6%, respectively, and one QTL (qSTR-17-CH14-A8–1), detected only in 2017, explaining 11.9% of PV.

#### Fiber elongation (ELO)

Nine QTLs explaining 5.7 to 13.5% of PV were mapped in the linkage maps (Table 5 and Additional file 1: Table S1). Of these 9 QTLs for elongation, four were major QTLs which included three stable QTLs (qELO-CH4-A11–1, qELO-CH8-A9–1, qELO-CH19-D1–1) with 7.1–13.5%, 7.5–12.3%, 8.1–12.4% of PVE and one QTL (qELO-17-CH23-A2–1) detected only in 2017, explained 11.2% of PV. Further, five of these mapped QTLs had favorable alleles from NC11–2091 for fiber elongation.

#### Short fiber content (SFC)

A total of 12 QTLs explaining 4.9 to 20.6% of PV were identified, including 5 major QTLs. One major QTL (qSFC-CH3-A3–1) was detected in both years with 7.9–18.4% of PVE, to which the favorable allele was contributed by NC05AZ06. Another 4 major QTLs (qSFC-17-CH2-A13–1, qSFC-16-CH4-A11–1, qSFC-17-CH5-D11–1, qSFC-17-CH18-A6–1) with the PVE ranging from 12.4 to 20.6% were detected in a single year environment. Since cotton fiber with high SFC is adverse to its quality [44], SFC with lower values are considered favorable. Most of the QTL favorable alleles were derived from NC05AZ06, except for qSFC-CH3-A3–1 and qSFC-17-CH26-D6–1.

### QTL for yield traits

A total of 38 QTLs, including 5 stable QTLs, 16 QTLs in 2016 and 17 QTLs in 2017, were identified for yield traits (BW, LP, SI, LI), with the PVE ranging from 4.2 to 30.4% (Table 6 and Additional file 1: Table S1). Accession NC05AZ06 contributed favorable alleles to 35 QTLs while NC11–2091 only donated favorable alleles for 3 of these 38 total QTLs. Further, of the 38 yield QTLs, 22 QTLs were mapped in the A sub-genome, including 19 QTLs with favorable alleles contributed by NC05AZ06 and 3 contributed by NC11–2091 and 16 QTLs were mapped in the D sub-genome, of which the favorable alleles were all contributed by NC05AZ06.

#### Boll weight (BW)

For boll weight, 11 QTLs explaining 5 to 14% of PV were identified and all favorable alleles of the QTLs were derived from NC05AZ06. Two major QTLs (qBW-16-CH4-A11–1, qBW-16-CH22-D13–1) with 14.0, 12.4% of PVE, respectively were detected only in year 2016 and another major QTL qBW-17-CH4-A11–1 with 12.1% of PVE was identified in year 2017.

#### Lint percentage (LP)

Eight QTLs explaining 5.9 to 17.7% of PV were identified for lint percentage (LP) which included 3 major QTLs (Table 6 and Additional file 1: Table S1). Two major and stable QTLs qLP-CH24-D3–1 and qLP-CH25-D12–1 explained 8.8–16.6%, 5.9–17.7% of PV, respectively for LP. Another major QTL (qLP-17-CH14-A8–1) with 15.2% of PVE, were detected in 2017 dataset. All favorable alleles of these QTLs were derived from NC05AZ06.

#### Seed index (SI)

For seed index, 9 QTLs explaining 5.8 to 30.4% of PV were detected. Among them, 6 QTLs with favorable alleles were derived from NC05AZ06. Two major and stable QTLs qSI-CH12-D10–1 and qSI-CH15-A12–1 with 27.1–30.4%, 10.6–17.5% of PVE, respectively, were identified in both environments.

#### Lint index (LI)

Ten QTLs explaining 4.2 to 21.1% of PV for lint index were identified and all their favorable alleles were contributed by NC05AZ06. Four major QTLs, including two QTLs detected only in the year 2016 (qLI-16-CH2-A13–1, qLI-16-CH12-D10–1) and other two QTLs detected only in year 2017 (qLI-17-CH22-D13–1, qLI-17-CH24-D3–1), explained 10.8, 13.5, 21.1, 12.6% of PV, respectively.

### QTL for morphological traits

A total of 9 QTLs, including 2 stable QTLs, 1 QTL in 2016 and 6 QTLs in 2017, were identified for morphological traits (plant height and fuzziness grade), with the PVE from 3.6 to 48% (Table 7 and Additional file 1: Table S1). Accession NC05AZ06 contributed favorable alleles to 2 QTLs (qFG-CH22-D13–1, qFG-16-CH25-D12–2) whereas NC11–2091 donated favorable alleles for 7 of the 9 total QTLs (4 QTLs on the A sub-genome and 5 QTLs on the D sub-genome).

#### Plant height (PH)

Five QTLs explaining 6.5 to 15.8% of PV were identified in year 2017 and all these QTLs with positive additive effect for plant height were derived from NC11–2091. Three major QTLs for plant height (qPH-17-CH8-A9–1, qPH-17-CH9-D5–1, qPH-17-CH19-D1–1) explained 10.3, 15.8 and 10.4% of PV, respectively (Table 7).

#### Seed fuzziness grade (FG)

For seed fuzziness grade, 4 QTLs explaining 3.6 to 48% of PV were identified, of which 2 are major stable QTLs. Major stable QTL (qFG-CH22-D13–1) was the only QTL with positive additive effect for seed fuzziness contributed by NC05AZ06, with 39.2–48% of PVE. Another major stable QTL (qFG-CH25-D12–1) explained 3.6–19% of PV for seed fuzziness (Table 7).

### QTL clusters

A QTL cluster is a short region (< 30 cM) on the linkage map containing multiple QTLs [14]. In this study, 21 QTL Clusters (Tables 8 and 9) were identified on 16 different chromosomes (Chr3, Chr4, Chr7, Chr8, Chr9, Chr10, Chr11, Chr12, Chr15, Chr16, Chr19, Chr21, Chr22, Chr23, Chr24 and Chr25) (Figs. 1, 2, 3, 4, 5, 6 and 7). Twelve QTL clusters were detected in A sub-genome and 9 clusters were detected in D sub-genome. Seven QTL clusters (Q-1 to Q-7) contained multiple fiber quality trait QTLs. Cluster Q-1, Q-3, Q-4, Q-5, Q-6 were identified with QTLs from SFC and UI (Table 8). In each of these 5 clusters, the favorable alleles of SFC and UI QTLs were contributed by same parents with different signs (“+” or “-”) of additive effects. For yield traits, four QTL clusters (Y-1 to Y-4) were identified (Table 8). The favorable alleles for most of the yield QTLs in these clusters were derived from NC05AZ06.

**Table 8:** Clusters containing multiple QTLs from same trait category (fiber quality or yield). QTL mapping study involved the RIL population of cross NC05AZ06 × NC11–2091 genotyping with Cotton 63K SNP array. Phenotypic traits were evaluated in the field at Central Crops Research Station, Clayton, NC in years 2016 and 2017

QTL Cluster^a^	Chromosome	Genetic map location	QTLs clustered	Trait	Source of favorable alleles	PVE
Q-1	CH3	46.82–59.15	qSFC-CH3-A3–1	SFC	NC11–2091	7.9
			qUI-CH3-A3–1	UI	NC11–2091	21
Q-2	CH7	17.16–27.41	qUHM-17-CH7-A7–1	UHM	NC05AZ06	10.1
			qELO-16-CH7-A7–1	ELO	NC11–2091	6.7
Q-3	CH10	12.09–19.17	qUI-16-CH10-A5–1	UI	NC05AZ06	12.7
			qSFC-16-CH10-A5–1	SFC	NC05AZ06	6
Q-4	CH11	30.34–52.37	qUI-CH11-A10–1	UI	NC05AZ06	16.1
			qSFC-16-CH11-A10–1	SFC	NC05AZ06	9.5
Q-5	CH21	11.56–18.62	qUI-17-CH21-D2–1	UI	NC05AZ06	10
			qSFC-CH21-D2–1	SFC	NC05AZ06	4.9
Q-6	CH22	68.73–79.76	qUI-17-CH22-D13–1	UI	NC05AZ06	9.5
			qSFC-17-CH22-D13–1	SFC	NC05AZ06	6.9
Q-7	CH23	133.16–142.33	qUHM-17-CH23-A2–1	UHM	NC05AZ06	10.2
			qELO-17-CH23-A2–1	ELO	NC11–2091	11.2
			qSFC-16-CH23-A2–1	SFC	NC05AZ06	6.4
Y-1	CH12	80.19–99.78	qLI-16-CH12-D10–1	LI	NC05AZ06	13.5
			qBW-17-CH12-D10–1	BW	NC05AZ06	8.6
			qSI-CH12-D10–1	SI	NC05AZ06	27.1
Y-2	CH15	19.71–38.68	qLP-17-CH15-A12–1	LP	NC05AZ06	6.1
			qSI-CH15-A12–1	SI	NC11–2091	17.5
Y-3	CH16	44.41–53.76	qBW-16-CH16-A1–1	BW	NC05AZ06	9.9
			qLI-16-CH16-A1–1	LI	NC05AZ06	6.8
			qSI-16-CH16-A1–1	SI	NC05AZ06	6
Y-4	CH22	91.66–103.29	qLI-17-CH22-D13–1	LI	NC05AZ06	21.1
			qBW-16-CH22-D13–1	BW	NC05AZ06	12.4

^a^Q: Cluster containing multiple QTLs for fiber quality traits; Y: Cluster containing multiple QTLs for yield traits

**Table 9:** Clusters containing multiple QTLs from different trait categories (fiber quality, yield or morphological). QTL mapping study involved the RIL population of cross NC05AZ06 × NC11–2091 genotyping with Cotton 63K SNP array. Phenotypic traits were evaluated in the field at Central Crops Research Station, Clayton, NC in years 2016 and 2017

Cluster^a^	Chr.	Region	QTLs	Trait	Source of the favorable alleles	PVE
QY-1	CH4	156.02–181.46	qELO-CH4-A11–1	ELO	NC11–2091	7.1
			qBW-17-CH4-A11–1	BW	NC05AZ06	12.1
QY-2	CH4	214.51–226.53	qBW-16-CH4-A11–1	BW	NC05AZ06	14
			qUI-16-CH4-A11–1	UI	NC05AZ06	13
			qSFC-16-CH4-A11–1	SFC	NC05AZ06	12.4
QY-3	CH7	126.63–139.78	qLI-CH7-A7–1	LI	NC05AZ06	4.2
			qMIC-16-CH7-A7–1	MIC	NC05AZ06	6.4
			qSI-17-CH7-A7–1	SI	NC05AZ06	7
QY-4	CH24	60.96–81.76	qUHM-17-CH24-D3–1	UHM	NC05AZ06	7.5
			qLI-17-CH24-D3–1	LI	NC05AZ06	12.6
			qLP-CH24-D3–1	LP	NC05AZ06	16.6
			qMIC-CH24-D3–1	MIC	NC05AZ06	25.8
QY-5	CH24	119.17–141.3	qSTR-17-CH24-D3–1	STR	NC11–2091	5.8
			qUHM-16-CH24-D3–1	UHM	NC05AZ06	11.9
			qLP-16-CH24-D3–2	LP	NC05AZ06	6.7
QA-1	CH7	48.29–61.65	qUHM-16-CH7-A7–1	UHM	NC05AZ06	12.1
			qUI-17-CH7-A7–1	UI	NC05AZ06	8.9
			qPH-17-CH7-A7–1	PH	NC11–2091	6.5
QA-2	CH19	124.72–136.4	qSTR-CH19-D1–1	STR	NC11–2091	5.2
			qPH-17-CH19-D1–1	PH	NC11–2091	10.4
			qELO-CH19-D1–1	ELO	NC05AZ06	12.4
YA-1	CH9	183.45–193.74	qPH-17-CH9-D5–1	PH	NC11–2091	15.8
			qLI-16-CH9-D5–1	LI	NC05AZ06	8.9
QYA-1	CH8	56.8–72.49	qPH-17-CH8-A9–1	PH	NC11–2091	10.3
			qELO-CH8-A9–1	ELO	NC05AZ06	12.3
			qLP-16-CH8-A9–1	LP	NC05AZ06	7.6
			qFG-17-CH8-A9–1	FG	NC11–2091	8.1
QYA-2	CH25	97.24–108.63	qLP-CH25-D12–1	LP	NC05AZ06	5.9
			qFG-CH25-D12–1	FG	NC11–2091	19
			qMIC-CH25-D12–1	MIC	NC05AZ06	4.1
			qSTR-CH25-D12–1	STR	NC11–2091	15.6

^a^QY- Cluster containing multiple QTLs for fiber quality and yield traits; QA- Cluster containing multiple QTLs for fiber quality and morphological traits; YA- Cluster containing multiple QTLs for yield and morphological traits; QYA- Cluster containing multiple QTLs for fiber quality, yield and morphological trait

Ten QTL clusters contained multiple QTLs from different trait categories (Table 9). The QYA-1 and QYA-2 were two clusters carrying multiple QTLs from all 3 trait categories. The QYA-1 with a region in Chr.8 from 56.8 cM to 78.78 cM, contained 4 QTLs for FG, ELO, LP and PH. QYA-2 with a region in Chr.25 from 97.24 cM to 108.63 cM, carried 4 QTLs for STR, MIC, LP and FG.

### Meta QTL analysis

A total of 2884 cotton QTLs for 11 traits: MIC(442), UHM(524), UI(289), STR(470), ELO(287), SFC(58), BW(176), LP(327), LI(42), SI(147), PH(122), which were collected by the CottonQTLdb [14, 15, 45] in different interspecific or intraspecific populations from 156 previous publications (http://www2.cottonqtldb.org:8081/references), were used for meta-QTL analysis in recent study (See additional file 3: Table S3).

In the current study, 74 QTLs were found to share the similar genetic positions (genetic distance window of < 20 cM) with previous reported QTLs, including 39 QTLs in the A sub-genome and 35 QTLs in D sub-genome. All these 74 shared QTLs were separated in to 11 different traits: STR (11), UI (10), UHM (7), MIC (7), ELO (7), SFC (7), BW (7), SI (6), LP (5), LI (4) and PH (3), including 33 major QTLs. Thirteen of these shared QTLs were stable QTLs (qELO-CH8-A9–1, qSTR-CH2-A13–1, qSTR-CH19-D1–1, qSTR-CH25-D12–1, qLP-CH24-D3–1, qMIC-CH10-A5–1, qMIC-CH24-D3–1, qMIC-CH25-D12–1, qSFC-CH3-A3–1, qSI-CH12-D10–1, qSI-CH15-A12–1, qUI-CH3-A3–1, qUI-CH11-A10–1). More than 70% of the QTLs shared the similar genetic positions with previously reported fiber quality and yield QTLs, which indicating consistency between the current study and previous studies. All the QTLs for STR, UI and MIC located on the similar genetic positions with previously reported QTLs. Twenty-eighty QTLs were unique QTLs with 17 QTLs in A sub-genome and 11 QTLs in D sub-genome, including 5 for SFC, 3 for UHM, 2 for ELO, 6 for LI, 4 for BW, 3 for SI, 3 for LP, 2 for PH. Out of these 28 unique QTLs, 11 were major QTLs (qBW-17-CH4-A11–1, qBW-16-CH4-A11–1, qELO-CH4-A11–1, qELO-CH19-D1–1, qLI-17-CH24-D3–1, qLI-16-CH12-D10–1, qLP-CH25-D12–1, qPH-17-CH9-D5–1, qSFC-17-CH2-A13–1, qUHM-17-CH23-A2–1, qUHM-16-CH24-D3–1). Three of them were stable QTLs: qELO-CH4-A11–1, qELO-CH19-D1–1, qLP-CH25-D12–1, which could be good addition to the existing QTLs.

### Candidate gene analysis

BLAST searching of the 22 genomic regions harboring stable QTLs in the Cotton Functional Genomics Database (https://cottonfgd.org/) identified 33 known genes as candidates genes that had been reported [46–58] to have functional role in cotton fiber development [59] (Additional file 5: Table S5). Out of these 33 candidate genes, 19 genes, reportedly have functional role in fiber development, were mapped in the 11 major and stable QTL regions which were identified in both years. These included 3 QTLs for ELO, 3 QTLs for STR, 2 QTLs for MIC, 1 for UI, 1 for SFC and 1 for LP (Table 10). Further, the 6 reported fiber related candidate genes were found in the QTL cluster QYA-2 on chromosome D12, which contained 4 major stable QTLs: qLP-CH25-D12–1, qMIC-CH25-D12–1, qSTR-CH25-D12–1 and qFG-CH25-D12–1 (Additional file 5: Table S5).

**Table 10:** Information of the reported cotton fiber related genes located in the major stable QTL regions identified in both 2016 and 2017

QTL	Chr	Known Genes	Name of gene in Genome	Position	Description of gene founction
qUI-CH3-A3–1	A03	GhMYB4	Gh_A03G1418	93,758,789–93,759,852	Preferential expression during cotton fiber development [47]
qSFC-CH3-A3–1					
qMIC-CH10-A5–1	A05	GhMYB5	Gh_A05G0291	3,355,753–3,356,568	Preferential expression during cotton fiber development [47]
qELO-CH8-A9–1	A09	GhPAG1	Gh_A09G1002	59,935,530–59,938,444	Playing a crucial role in regulating fiber development [49]
		GhMYB1	Gh_A09G1008	60,122,091–60,128,474	Preferential expression during cotton fiber development [47]
		GhPAG1	Gh_A09G1009	60,136,650–60,139,807	Playing a crucial role in regulating fiber development [49]
		GhTCP11	Gh_A09G1389	67,016,702–67,017,304	Preferentially expressed in cotton fibers at the stage of secondary cell wall biosynthesis [48]
qELO-CH4-A11–1	A11	GhCPC	Gh_A11G0869	8,849,618–8,850,249	Negatively regulating cotton fiber initiation and early elongation [50]
		GhPRP3	Gh_A11G1028	11,619,507–11,620,113	Potentially as a negative regulator participating in modulating fiber development of cotton [51]
		GhMYB4	Gh_A11G1203	14,890,234–14,891,570	Preferential expression during cotton fiber development [47]
qSTR-CH2-A13–1	A13	GhMYB5	Gh_A13G0805	37,876,373–37,877,410	Preferential expression during cotton fiber development [47]
		GhTCP15	Gh_A13G0648	18,141,353–18,142,453	Preferentially and predominantly expressed in fast elongating fibers [48]
qELO-CH19-D1–1	D01	Gh14–3-3	Gh_D01G0107	823,416–825,377	May involving in regulating fibre initiation and elongation [52]
qSTR-CH19-D1–1		GhMYB4	Gh_D01G0155	1,139,298–1,142,633	Preferential expression during cotton fiber development [47]
qSTR-CH25-D12–1	D12	GhPIP2–4	Gh_D12G1974	52,804,569–52,815,428	Involving in cotton fibre development by regulating water channel activities [58]
qMIC-CH25-D12–1 qSTR-CH25-D12–1 qLP-CH25-D12–1	D12	GhTCP5	Gh_D12G1814	50,669,259–50,670,242	Preferentially expressed in secondary cell wall deposition stage [48]
		GhTCP14	Gh_D12G1742	49,671,539–49,672,741	Play critical roles in cotton fiber development expressed predominantly in initiating and elongating fibers [48]
		GhTCP12	Gh_D12G1689	48,768,374–48,769,879	preferentially expressed in cotton fiber initiation and secondary cell wall deposition stage [48]
		GhTCP15	Gh_D12G1644	47,951,010–47,952,044	Preferentially and predominantly expressed in fast elongating fibers [48]
qMIC-CH25-D12–1	D12	GhTCP20	Gh_D12G1425	43,870,059–43,870,949	preferentially expressed during cotton fiber development [48]
qLP-CH25-D12–1					

## Discussion

### Construction of high-density linkage maps with SNP arrays

The limited quantity of the polymorphic markers available were often limitations for the construction of high-density linkage maps in cotton [60, 61]. Due to the lack of the marker polymorphism in cotton, the linkage maps built by second-generation molecular markers such as SSRs and AFLPs, usually carried some disadvantages viz low marker coverage of the cotton genome, poor marker density and large gaps [61–64]. SNPs provide abundant genetic variation and their loci distribute evenly along the whole genome. Hence, they have been the most reliable markers for building high-density linkage maps and have been widely used in the QTL studies [40, 65, 66]. Recently, two sets of cotton SNP arrays CottonSNP63K and CottonSNP80K were developed and were used in the QTL mapping [40, 65]. Several high-density cotton genetic maps constructed successfully using these SNP arrays [35, 66–70]. In the current study, a linkage map was constructed using SNPs from CottonSNP63K array. The genetic map spanned a total length of 4422.44 cM, which was in correspondence with the estimated size of tetraploid cotton genome (4500 cM) [71]. The average marker density of the map was 1.41 cM. No large gaps (> 15 cM) were found and marker density and coverage was better than the SSR-based linkage maps developed previously [27, 28, 30, 62–64, 72]. We identified 11,255 (17.8%) polymorphic SNPs between the parents from the 63,058 SNP markers and only 3129 (5.0%) of the polymorphic SNPs were unique. Based on the previous studies, the polymorphism rate of SSRs and SNPs for a RIL population was around 3–10% [62–69]. Out of a total 3129 SNP markers mapped, distribution of SNP markers was fairly even between A (1,534 SNPs) and D (1,595 SNPs) sub-genomes. Genetic map lengths produced by these markers in A and D sub-genomes were 2236.35 cM and 2186.09 cM, respectively. Further, SNP linkage maps showed a high level of collinearity with the sequence based physical map of Upland cotton (Fig. 8) suggesting there were no chromosome rearrangements among the parents and mapping population used for mapping. Finally, the circos plot suggested the accuracy of the linkage maps in comparison to the physical maps. Circos plots further confirmed that the polymorphic SNPs detected in each of the chromosomes distributed unevenly, which support an observation that the SNPs showed uneven distribution for polymorphism-rich and polymorphism-poor regions along each chromosome [73].

### Segregation distortion

Segregation distortion (SD), commonly observed in mapping populations [33, 35, 66, 67, 69], could be due to genetic drift, preferential fertilization by particular gametic combinations and due to environmental factors [74–76]. In this study, 5.6% (175) of the mapped markers, showed segregation distortion (Table 4). This was lower than the previous reports in cotton (11.4–32.8%). Wang et al. [33] reported that the bigger the genetic differentiation between two parents, the smaller the segregation distortion in the derived population. This may suggest lower SD in our study since the parents used in this study were expected to show maximum allele diversity. Fifty-nine percent of distorted markers (103) were on 6 chromosomes (A3, A10, A12, A13, D5, D10), which was consistent with the previous reports that the majority of distorted markers were concentrated in a few chromosomes [33, 35, 66, 67, 69, 74–76].

### QTL mapping population

The quality of a QTL map depends on the number of polymorphic markers and the genetic mapping populations used. Tetraploid cotton, in general, show a low level of marker polymorphism [77, 78]. According to the previous cotton QTL mapping research, it was observed that the marker polymorphism rates in the interspecific mapping populations [24, 31, 32, 72, 79, 80] were higher than intraspecific mapping populations [35–39] on the whole. In order to potentially detect a broader array of polymorphic markers and QTL alleles, interspecific mapping populations derived from *G. hirsutum* and *G. barbadense* have been extensively used for QTL mapping in cotton [24, 31, 32, 72, 79, 80]. However, the QTL type and their mapping information from an interspecific (*G. hirsutum* × *G. barbadense*) population were inconsistent in comparison to the QTLs studied based on an intraspecific *G. hirsutum* population [15]. Further, QTLs identified using the interspecific RILs could not be transferred precisely into Upland cotton due to the genetic bottlenecks associated with interspecific hybridizations during the breeding process. Hence, QTLs of the interspecific mapping studies were not utilized in Upland cotton improvement. In this study, an intraspecific *G. hirsutum* RIL population, developed from a cross between an improved germplasm line NC05AZ06 and a landrace accession NC11–2091 was used for QTL mapping. The CottonSNP63K array based genotyping provided good number of the candidate markers. This allowed us to obtain enough polymorphic markers to develop high density genetic maps in Upland cotton which in general suffers from low density of markers and low marker polymorphism [24, 31].

### QTLs with favorable alleles identification

The identification of favorable QTLs alleles can help improving the fiber quality and yield in Upland cotton by genomic and marker assisted selection [81]. As expected, the performance of parent NC05AZ06 was superior to those of the landrace parental accession NC11–2091 for MIC, UHM, UI, STR and 4 yield traits. Among the 106 QTLs, the favorable alleles of 80 QTLs originated from NC05AZ06 while other 26 from NC11–2091. Only a few of the QTLs with favorable alleles of a given trait were derived from the parent NC11–2091 (Table 7 and Additional file 1: Table S1). Fifteen QTLs with favorable alleles contributed by NC11–2091. Of these, 8 were major QTLs.

### QTL locations and clusters

Based on the reports from previous cotton QTL studies (http://www2.cottonqtldb.org:8081/references) [45], the QTLs for fiber quality traits and yield traits were distributed on most chromosomes, varied from population to population (See Additional file 3: Table S3). Of the 44 major QTLs for the 11 traits in the current study, eleven QTLs were unique QTLs: 2 for ELO (qELO-CH4-A11–1, qELO-CH19-D1–1), 2 for UHM (qUHM-17-CH23-A2–1, qUHM-16-CH24-D3–1), 1 for SFC (qSFC-17-CH2-A13–1), 2 for BW (qBW-17-CH4-A11–1, qBW-16-CH4-A11–1), 2 for LI (qLI-17-CH24-D3–1, qLI-16-CH12-D10–1), 1 for LP (qLP-CH25-D12–1) and 1 for PH (qPH-17-CH9-D5–1). The presence of the unique QTLs was expected because of the type of parental accessions used and the number of the SNP markers used to detect the maximum allele diversity. Out of these unique QTLs,11 were major QTLs, 3 were stable QTLs (qELO-CH4-A11–1, qELO-CH19-D1–1, qLP-CH25-D12–1). Most of the QTLs were detected on the chromosomes that were shown to carry the QTLs for the corresponding traits (See Additional file 1: Table S1 and Additional file 3: Table S3). Only 5 major QTLs were detected on the chromosomes where there were no previously reported QTLs for the corresponding traits: qBW-16-CH4-A11–1(A11), qLI-17-CH24-D3–1(D03), qLI-16-CH12-D10–1(D10), qSFC-17-CH2-A13–1(A13), qUHM-17-CH23-A2–1(A2) (See Additional file 1: Table S1). Huang et al. 2017 [82] reported a genome-wide association study (GWAS) in Upland cotton using the CottonSNP63K array. Twelve QTLs mapped in the current study showed similar physical position with the QTLs reported by Huang et al. 2017 [82] for the identical traits (Table 11). Of the 4 stable QTLs identified in the current study (qUI-CH3-A3–1, qUI-CH11-A10–1, qLP-CH25-D12–1, qMIC-CH25-D12–1), the QTL for LP and MIC in the QTL cluster on D12 showed similar chromosome location as were reported by Huang et al. 2017 [82]. Identification of this QTL cluster from independent studies involving diverse mapping populations validates and proves the QTL region on D12 for fiber quality traits. These could be potential targets for MAS and map-based cloning of major fiber quality QTLs in Upland cotton.

**Table 11:** List of the QTLs locating in similar physical position with the QTLs reported by Huang et al. for the identical traits [82]

Trait^a^	QTL	Chromosome	Physical position	Referenced QTL^b^
UI	qUI-CH3-A3–1	A03	94,326,661–94,875,918	qGhFU-c3-A3
LP	qLP-16-CH8-A9–1	A09	62,770,225–65,707,079	qGhLP-c9-A9
UI	qUI-CH11-A10–1	A10	85,538,436–92,041,525	qGhFU-c10-A10–1
LI	qLI-17-CH4-A11–1	A11	77,114,278–81,492,822	qGhLW-c11-A11
FE	qFE-16-CH20-D4–1	D04	47,939,786–48,742,306	qGhFE-c22-D4–1
LI	qLI-16-CH9-D5–1	D05	8,708,185–9,529,637	qGhLW-c19-D5
SFC	qSFC-16-CH9-D5–1	D05	11,211,267–12,030,834	qGhSF-c19-D5–3
LI	qLI-17-CH17-D8–1	D08	29,437,993–38,988,165	qGhLW-c24-D8–2
LI	qLI-16-CH12-D10–1	D10	12,408,822–16,285,673	qGhLW-c20-D10
UHM	qUHM-17-CH5-D11–1	D11	21,238,116–24,240,808	qGhFUHML-c21-D11–1
LP	qLP-CH25-D12–1	D12	38,582,591–42,990,683	qGhLP-c26-D12–1
MIC	qMIC-CH25-D12–1	D12	44,214,339–48,629,081	qGhMV-c26-D12–1

^a^ *MIC* micronaire, *UHM* upper half mean length, *UI* uniformity index, *SFC* short fiber content, *LP* lint percentage, *LI* lint index;

^b^ Names of the QTLs reported by Huang et al.

Many genetic studies on cotton seed fuzzless trait have been carried out previously. In 1949, Ware et al. [83] first studied this seed fuzzless character and reported it was controlled by a single gene. But later, other reports suggested it was not a binary trait of naked or fuzzy seed, but there existed different degrees of seed fuzziness performance which may be controlled polygenically [10, 11, 13]. Previous study reported that there were two seed fuzzless trait loci on chromosomes A12 and D13 which were controlled by major genes [11]. Our results not only confirmed the genetic factors located on D13, but also identified a new locus on D12 for seed fuzzless trait (Table 7 and Additional file 1: Table S1). It is interestingly to note that the new locus (qFG-CH25-D12–1) was mapped on chromosome D12 which is homoeologous chromosome A12, previously reported [11] to carry fuzzless trait suggesting the functional conservation of orthologous genomic regions controlling the fuzzless trait in Upland cotton. Majority of the QTLs showing shared position with previous studies suggest the genetic relatedness of the elite cottons of the USA and in general narrow genetic base of cultivated cotton. This further indicates that the marker trait associations identified for quantitatively inherited cotton traits could be broadly applicable across most cotton breeding programs.

The phenotypic trait correlation analysis showed high values of positive or negative correlations between different traits, which can be partially explained by the QTL clusters we identified. For example, Q-1, Q-3, Q-4, Q-5, Q-6 contained multiple QTLs from SFC and UI (Table 8). However, the signs of additive effects of SFC and UI QTLs in each of these clusters were opposite with favorable alleles from same parent. In this case, when we choose the favorable alleles of NC05AZ06 for this QTL, SFC will decrease and UI will increase. If we choose the other alleles for the QTL, the UI will decrease and SFC will increase. This explained why UI and SFC would always show negative correlation values (− 0.93). This strong negative relationship between SFC and UI was also reported in the previous study by Ramey et al. [84]. On the contrary, Y-1, Y-3, Y-4 clusters provided the evidence of why all the yield traits were highly positively correlated since all the favorable alleles of QTLs in these clusters were derived from same parent. These positive correlations among yield traits were also widely observed in many previous studies [31–33, 35, 37, 66, 67]. Similarly, Q-2, Q-7 explained a negative correlation (− 0.62) between UHM and ELO. This is consistent with previous observation by Wang et al. [33] who reported a negative correlation (− 0.59) between fiber length and ELO. Q-7 also explained a positive correlation (0.46) between ELO and SFC as well as a negative correlation (− 0.79) between UHM and SFC. Interestingly, previous reports suggested both positive [85] as well as negative correlation (− 0.349) [61] between ELO and SFC. In the current study, some of the clusters contained both fiber quality traits and yield traits, which provided us an efficient way to improve the quality traits and yield traits at the same time. For example, QY-3 were shared by 3 QTLs from LI, SI and MIC, of which the favorable alleles were all contributed by NC05AZ06. In this case, the QTL markers in QY-3 can help improving the MIC, LI and SI concurrently. Similarly, the QTLs in QY-4 had the potential to improve the UHM, MIC, LI and LP simultaneously. Similar type of positive correlation between LI, LP and MIC was reported by Wang et al. [33].

### Candidate gene analysis of the QTLs

The identification of the candidate genes with known functions in cotton fiber development, located in the mapped QTL regions, could add additional validity to the fiber quality QTLs. Out of the 11 stable and major QTLs analyzed, 8 regions with QTLs qELO-CH8-A9–1, qELO-CH4-A11–1, qSTR-CH2-A13–1, qELO-CH19-D1–1, qSTR-CH19-D1–1, qSTR-CH25-D12–1, qMIC-CH25-D12–1, qLP-CH25-D12–1 showed two or more cotton fiber related candidate genes (Additional file 5: Table S5). Moreover, a fiber related gene-rich QTL cluster QYA2 was identified on chromosome D12. The presence of 6 reported fiber related candidate genes localized in this QTL region may partially explain and confirm the QTL cluster containing multiple different QTLs. The importance and validation of this QTL on chromosome D12 could also be confirmed from the previous mapping efforts. Huang et al. 2017 [82] reported a genome-wide association study (GWAS) in Upland cotton using the CottonSNP63K array and performed the BLAST search using the SNPs underlying QTLs against the Genome NAU-NBI v1.1 database [82]. The QTL for LP (qLP-CH25-D12–1) and MIC (qMIC-CH25-D12–1) in the QTL cluster on D12 showed similar chromosome location and candidate genes as were reported by Huang et al. 2017 [82]. Identification of QTL clusters from independent studies involving diverse mapping populations validates and proves the QTL regions. Such QTLs could be potential targets for MAS and map-based cloning of major fiber quality QTLs in Upland cotton.

## Conclusions

A high-density linkage map spanning 4422.44 cM length with an average marker density of 1.41 cM was developed using 3129 SNP markers. Genetic maps showed high level of collinearity with their corresponding sequence based physical maps. Forty-six major QTLs were identified with 29 QTLs for fiber quality traits, 12 for yield traits and 5 for morphological traits. More than 70 % of the mapped QTLs shared the similar linkage and physical position with previously reported QTLs. QTLs for fiber quality showed clustering on a handful of chromosomal regions indicating these are possible regions of major selective sweeps, which could help explain the strong correlation between fiber quality traits in cotton. Majority of the QTLs showing shared position with previous studies suggest that the genetic relatedness of the elite cottons of the USA and the general narrow genetic base of cultivated cotton. Candidate gene analyses of the stable QTLs identified candidate genes with functional roles in fiber development. The stable QTLs, major QTLs and the QTL clusters identified in the SNP map in the current study could be the potential targets for MAS in cotton breeding and map-based cloning of QTLs controlling fiber quality traits in cotton.

## Methods

### Development of the RIL population

The *G. hirsutum* accessions NC05AZ06 and NC11–2091 were used as parents to develop the RIL population. NC05AZ06 is a sub-okra germplasm line with improved fiber quality and yield traits released by our program [86]. The landrace accession NC11–2091(TEX 2313; PI 607640), collected from Thailand, was obtained from the U.S. National Cotton Germplasm Collection (NCGC), USDA-ARS, College Station, Texas. As landraces tend to be heterogeneous, we inbred the land race accession NC11–2091 for three generations using manual selfing and single seed descent method of line advancement. In the summer of 2010, the inbred parental accessions were planted at the Central Crops Research Station at Clayton, North Carolina and crossed to develop F_1_ seeds. The F_1_ plants were planted in the winter nursery, Tecoman, Mexico and manually selfed using glassine bags to obtain F_2_ seed. The F_2_ plants were grown and individual plants were manually selfed to obtain F_3_ seed in the summer nursery of 2012. From 2013 to 2015, 107 F_2:3_ lines were advanced to F_5_ generation by single seed decent method in the greenhouses. The 107 F_5:6_ lines were grown in the summer nursery at Clayton, NC in 2015 and seed increased by manual self-pollination. Seed cotton samples were ginned using 10-saw gin. Seed were acid delinted and treated with fungicide and insecticide before using in the current study.

### Field experiments and phenotyping

The F_5:6_ RIL population containing 107 RILs along with parents and four checks (UA-48, UA-222, DP-393, SG-747) were planted using an augmented randomized complete block design with seven blocks in Clayton, NC in summer 2016. Each line was planted (2.5–3 seeds per ft) in the single row 10-ft plots with 38-in row spacing and 10 ft. alleys. Standard morphological practices were followed. Fifty fully opened bolls from each plot were hand-harvested in November of each year of the trials. Four yield traits, including boll weight (BW), lint percentage (LP), seed index (SI), lint index (LT) were evaluated. Approximately 15 g (g) of fiber sample ginned from each boll sample was tested for the fiber quality parameters using high-volume instrument (HVI) system at the Cotton Incorporated, Cary, North Carolina. The fiber quality traits evaluated were fiber elongation (ELO), micronaire (MIC), short fiber content (SFC), fiber strength (STR), upper half mean length (UHM) and uniformity index (UI). MIC is an airflow measurement of fibers and indicates fiber fineness and maturity. UHM is the mean length of the longer half of the fibers in the sample, measured in hundredths of an inch. STR is the force in grams required to break a bundle of fibers one tex unit in size. ELO is the amount in percentage a fiber sample can stretch prior to breakage. UI is a ratio between the mean length and the upper half mean length of the fibers, expressed as a percentage. It indicates the uniformity of fiber length in a sample. SFC is the percentage by weight of fibers 0.5 in. (12.7 mm) long or less. BW is the average weight in grams of seedcotton in a boll. LP is a ratio between the total fiber weight and the total seedcotton weight. SI is the weight of 100 seeds in grams. LI is the weight of lint in grams obtained from 100 seeds. The morphological trait, fuzziness grade of seed (FG) was determined by rating based on four levels of the seed fuzziness (0, 33.3, 66.6 and 100%). Progressive numbers 0 to 100% indicate fuzz-free to fuzz-rich cotton seed.

In the summer of 2017, the RIL population along with parents and the same four checks were planted using a completely randomized block design (RCBD) with two replications in Clayton, NC. Each line was planted in the single row 20-ft plot with a plant density of 2.5 seeds per foot. Forty fully opened bolls from each plot were hand-harvested in December 2017. Same phenotyping methods were used for evaluating the 11 cotton traits as in year 2016. Plant height (PH) trait values was evaluated by taking the average of the manually-measured height of five randomly selected plants from each plot.

### Marker genotyping and linkage map construction

Genomic DNA was extracted from 3 to 4 weeks old plant leaf tissue of the RIL population and their parents using DNeasy Plant Mini Kit (Qiagen, Hilden, Germany). One hundred and four of the 107 phenotyped RILs and the parents were genotyped with 63 K cotton SNP array [41] at Texas A&M Institute for Genome Sciences and Society. Candidate SNPs were filtered from the array with 63,058 SNPs based on the rules as follows: (1) SNPs with monomorphic genotypes were removed, (2) poor-quality SNPs and SNPs with missing values more than 30% were removed and (3) duplicate SNPs were removed [67].

The resultant candidate SNPs were used to construct the linkage map by JoinMap 4.1 [87] using Kosambi’s mapping function [88] with logarithm of the odds (LOD) threshold of 7.0. The SNPs were then aligned to the TM-1 (*G. hirsutum*) Genome NAU-NBI Assembly v1.1 and Annotation v1.1 database [43] by BLAST (https://www.cottongen.org/blast). Correspondence of the linkage map groups with the physical map groups was performed with the circos plots by Circa software (http://omgenomics.com/circa/).

### Data analysis

All the trait phenotypic values of the RILs and parents were estimated using the linear mixed models in SAS version 9.4 (SAS Institute, Cary, NC). The SAS software was also used for calculating the statistics, including the T-test of the difference between the value means of two parents, the broad-sense heritabilities of the traits, the genetic correlations between the traits and other basic statistical parameters.

Segregation of the markers from the Mendelian ratio 1:1 was tested using chi-square analysis (*P* < 0.05) and a segregation distortion region (SDR) was identified when at least three adjacent markers showing significant (*P* < 0.05) segregation distortion [89] using JoinMap 4.1.

All 12 traits related QTLs were detected using composite interval mapping (CIM) method [90] using WinQTLCart2.5 [91]. The genotype of alleles from parental accession NC05AZ06 (P1) was coded as “AA” and the genotype of alleles from accession NC11–2091(P2) was coded as “aa”. Based on the results of a 1000-time permutation procedure, logarithm of the odds (LOD) ≥ 2.5 with at least 1 year’s phenotypic variation explained (PVE) ≥ 5.0 was used as the threshold for a QTL identified in both years with overlap region and LOD ≥ 3.0 with PVE ≥ 5.0 was the threshold to determine a QTL detected only in 1 year. The resulting linkage map with identified QTLs were drawn using MapChart version 2.32 [92]. Further, the identified QTLs were used to detect the QTL clusters and meta QTL analysis was performed by comparing them with the QTLs reported in previous studies. Information of all the previously reported QTLs was downloaded from the CottonQTLdb database (http://www.cottonqtldb.org; Release 2.3) developed by Said et al. [45]. The marker defined QTL regions with DNA sequence information were BLAST searched on Cotton Functional Genomics Database (https://cottonfgd.org/) [59] for identifying the possible candidates genes for the each of the major stable QTL.

### QTL nomenclatures

All the QTLs were labeled based on their population, trait type, and chromosome information. For example, QTLs for micronaire in population NC06AZ06 × NC11–2091 were labeled as qMIC-CH*-A(D)*-* (detected in both year), qMIC-16-CH*- A(D)*-* (detected in year 2016) or qMIC-17-CH*- A(D)*-* (detected in year 2017).

The names of the QTL clusters are given based on the trait categories of QTLs they contained. For example, Q-* meant a cluster contained only fiber quality traits QTLs; QY-* meant a cluster contained both fiber quality and yield traits QTLs; QYA-* meant a cluster contained fiber quality, yield and morphological traits QTLs and so on.

## Supplementary information

**Additional file 1: Table S1.** QTLs of fiber quality, yield and morphological traits identified in the RIL population.

**Additional file 2: Table S2.** Genetic and physical mapping details of the 3129 SNPs mapped in the current study.

**Additional file 3: Table S3.** Information of 2884 previously reported QTLs for the eleven traits.

**Additional file 4: Table S4.** Correspondence of linkage maps developed in the current study with sequence based physical maps of Upland cotton.

**Additional file 5: Table S5.** Candidate genes of cotton fiber quality and production and their bioinformatics descriptors identified for the QTL regions BALST searched in the cotton functional genomics database (https://cottonfgd.org/).

## Data Availability

The datasets supporting the findings of this article are included within the article and its additional files. Additional data used or analyzed during this study is also available from the corresponding author on reasonable request.

## Abbreviations

AE: Additive effect
AFLP: Amplified fragment length polymorphism
ARS: AGRICULTURAL Research Service
BW: Boll weight
CIM: Composite interval mapping
cM: centimorgan
DNA: Deoxyribonucleic acid
ELO: Fiber elongation
FG: Seed fuzziness grade
H^2^: The broad-sense heritability
InDels: Insertion-deletion polymorphisms
ISSR: Inter-simple sequence repeat
LI: Lint index
LOD: Logarithm of the odds
LP: Lint percentage
MAS: Maker-assisted selection
MIC: Micronaire
NCGC: U.S. National Cotton Germplasm Collection
NGS: Next-generation sequencing
PH: Plant height
PV: Phenotype variance
PVE: Phenotypic variation explained
QTL: Quantitative trait locus
RAPD: Random amplified polymorphic DNA
RFLP: Restriction fragment length polymorphism
RIL: Recombinant inbred line
SD: Standard deviation
SDR: Segregation distortion region
SFC: Short fiber content
SI: Seed index
SNP: Single nucleotide polymorphism
SRAP: Sequence related amplified polymorphism
SSR: Simple sequence repeats
STR: Fiber strength
TRAP: Target region amplified polymorphism
UHM: Upper half mean length
UI: Uniformity index
V_g_: Genetic variance
V_p_: Phenotypic variance
